# The Hippo pathway in oral diseases and treatments: A review

**DOI:** 10.1097/MD.0000000000040553

**Published:** 2024-11-08

**Authors:** Da Ni

**Affiliations:** a Stomatology Hospital, School of Stomatology, Zhejiang University School of Medicine, Zhejiang Provincial Clinical Research Center for Oral Diseases, Key Laboratory of Oral Biomedical Research of Zhejiang Province, Cancer Center of Zhejiang University, Engineering Research Center of Oral Biomaterials and Devices of Zhejiang Province, Hangzhou, China.

**Keywords:** Hippo pathway, oral diseases

## Abstract

This review aims to provide a recent update on the Hippo pathway in oral diseases. The Hippo pathway plays a crucial role in organ development, tissue regeneration, wound healing, maintaining epithelial homeostasis, and modulating the immune system. Globally, billions of people suffer from various oral diseases, posing significant public health risks and resulting in substantial economic losses. This article reviews the recent advancements in the research on the Hippo signaling pathway and its effectors in various conditions related to oral health. The implications of Hippo signaling in various dental fields, including endodontics, orthodontics, periodontology, oral implantology, oral and maxillofacial surgery, and oncology are discussed. It provides readers with an overview of the regulatory role of the Hippo pathway in the development of various oral diseases and the potential for exploiting this pathway for developing targeted therapeutics.

## 1. Introduction

The Hippo pathway was first discovered in *Drosophila*. After decades of research, a substantial amount of literature has reported that the Hippo signaling pathway plays crucial roles in various physiological and pathological processes, such as organ size determination, tissue regeneration, tumor growth, inflammation, and regulating stem cell biological behavior.^[1,2]^

Oral diseases affect approximately 3.6 billion people worldwide, resulting in economic losses amounting to hundreds of millions.^[3,4]^ Moreover, oral health is closely linked to a variety of systemic diseases, significantly influencing personal health and quality of life impacting individual health and quality of life.^[5]^ The oral cavity includes a variety of tissues, such as teeth, periodontal tissues, mucosa and connective tissues, salivary glands, and so on. They can suffer from damage, inflammation, malignancies, and other diseases under certain circumstances. Understanding the intrinsic molecular mechanisms behind the development of these diseases is vital for the prevention and treatment of these conditions.

In this review, we will focus on discussing recent advances of the regulatory role of the Hippo signaling pathway in various oral tissues, including its physiological and pathological effects, as well as potential research directions for promoting oral tissue health by utilizing the regulatory functions of the Hippo signaling pathway.

## 2. Brief introduction of Hippo pathway

The core of Hippo pathway consists of 4 tumor suppressors, namely Hippo (Hpo), Salvador (Sav), Warts, and Mob as tumor suppressor (Mats).^[6,7]^ These 4 proteins constitute a kinase cascade. Together with transcriptional coactivator Yorkie and transcription factor Scalloped (Sd), they regulate tissue growth in flies. The pathway was named after the Ste20-like protein kinase Hippo, because its mutation leads to gigantic organs in flies that seemed like the animal hippopotamus.^[8]^ The Hippo pathway is highly conserved from *Drosophila* to mammals. The mammalian Hippo pathway cascade consists of the proteins mammalian sterile 20-like kinase 1/2 (MST1/2) (Hippo [Hpo] ortholog), Salvador homolog-1 (SAV1) (Sav ortholog), large tumor suppressor 1/2 (LATS1/2) (Wts ortholog), Mps One Binder kinase activator protein 1 (MOB1) (Mats ortholog), Yes associated protein/transcriptional coactivator with PDZ-binding motif (YAP/TAZ) (Yki ortholog) and TEA domain family member1/2/3/4 (Sd ortholog) (Table 1).

**Table 1:** Components of Hippo pathway in *Drosophila* and mammals.

In *Drosophila*	Ortholog in mammals
Hippo (Hpo)	Mammalian sterile 20-like kinase1/2 (MST1/2)
Salvador (Sav)	Salvador homolog-1 (SAV1)
Warts (Wts)	Large tumor suppressor1/2 (LATS1/2)
Mob as tumor suppressor (Mats)	Mps One Binder kinase activator protein 1 (MOB1)
Yorkie (Yki)	Yes associated protein/transcriptional coactivator with PDZ-binding motif (YAP/TAZ)
Scalloped (Sd)	TEA domain family member (TEAD1/2/3/4)

**Figure 1.**
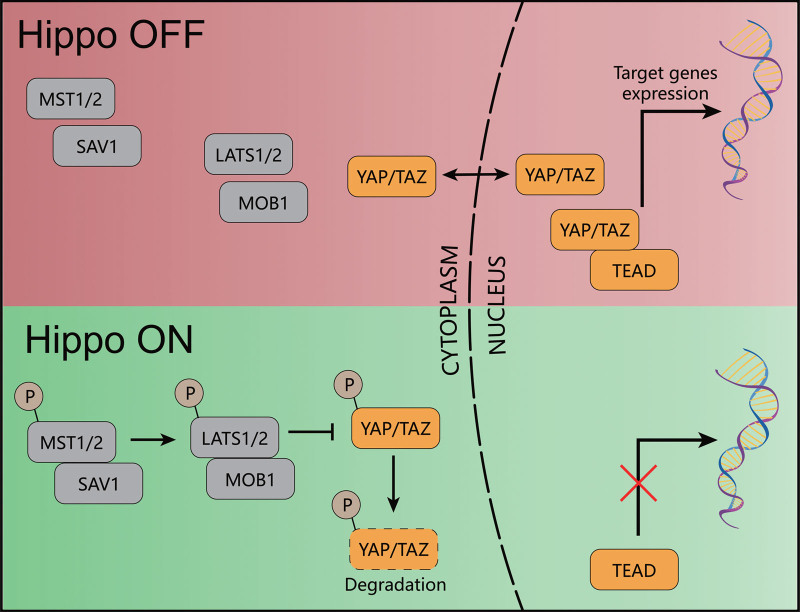
Illustration of core components of Hippo pathway.

When the Hippo pathway is activated, MST1/2-SAV1 heterodimers, which are phosphorylated, phosphorylate and activate the LATS1/2-MOB1A/B complex. This complex then phosphorylates YAP/TAZ which prevents their nuclear translocation and target gene expression. Phosphorylated YAP/TAZ either undergoes proteasome-mediated degradation or 14-3-3 protein-mediated cytoplasmic retention. In contrast, when the Hippo pathway is off, YAP/TAZ are dephosphorylated and translocated into the nucleus. YAP/TAZ do not bind to DNA, instead, they primarily bind to TEA domain family member and then initiate target gene transcription (Fig. 1).^[1,9,10]^

## 3. Hippo pathway in endodontics

Dental defects often lead to infection or inflammation of the dental pulp. Among them, reversible pulpitis can heal after treatment, whereas more severe pulp inflammation requires interventions like root canal treatment that result in the tooth losing its vitality and fragility. Vital dental pulp can continuously repair and regenerate dentin, which is crucial for maintaining the homeostasis and strength of dental tissues. Therefore, promoting the regeneration of dental pulp and dentin holds significant research value and has become a prominent topic in related fields. Most studies are centered on harnessing the migration, proliferation, and differentiation capabilities of dental pulp stem cells (DPSCs) to regenerate and repair dentin tissues effectively.

The Hippo pathway may have a controversial function on DPSCs. Du et al discovered through in vitro experiments that the long noncoding RNA H19 recruits the enzyme EZH2 to the promoter region of the *LATS1* gene. This recruitment leads to the trimethylation of histone 3 at lysine 27, thereby inhibiting the expression of *LATS1*. The loss of *LATS1* increases the levels of YAP/TAZ, which subsequently enhances the differentiation and proliferation of odontoblasts, as well as the migration capabilities of DPSCs.^[11]^

Similarly, Zheng et al found that a static magnetic field can affect the cell cytoskeleton, inhibit YAP/TAZ phosphorylation, and promote their nuclear translocation. This leads to increased expression of connective tissue growth factor and ankyrin repeat domain protein 1. Additionally, it enhances the expression of fibroblast growth factor 2, transforming growth factor β, and vascular endothelial growth factor, thereby promoting the proliferation, migration, osteogenesis, and odontogenesis capabilities of DPSCs.^[12]^ Furthermore, Tian et al found that silencing TAZ, the paralog of YAP, can inhibit the expression of connective tissue growth factor and cysteine-rich angiogenic inducer 61 through the transforming growth factor β pathway, ultimately suppressing the proliferation and migration of DPSCs.^[13]^ These studies indicate that YAP promotes the proliferation, migration, and differentiation of DPSCs.

Conversely, other studies suggest that YAP directs the cell fate of DPSCs toward stemness maintenance and inhibition of YAP leads to differentiation. An in vivo study reported that inhibiting the oxytocin receptor increases the level of phosphorylated LATS (pLATS) and phosphorylated YAP (pYAP), and reduces the nuclear localization of YAP, promoting osteogenic and odontogenic differentiation.^[14]^ Melatonin can induce the phosphorylation of YAP, reduce YAP expression, promote neural differentiation of DPSCs, and inhibit the proliferation of DPSCs.^[15]^

The transcriptional activity of YAP/TAZ can be regulated by mechanical signals also. A novel digested dentin matrix extract material was found to stimulate DPSCs, leading to the translocation of YAP into the nucleus. This enhances the expression of dentin matrix protein 1, collagen type 1 alpha 1 chain and runt-related transcription factor 2 (RUNX2), and enhances the regeneration of dentin.^[16]^ Other studies also suggest that YAP nuclear translocation can be influenced by morphological features of the substrate, such as pore size, roughness, and arrangement.^[17,18]^ Interestingly, a study reported that the rigidity of the substrate does not affect the intracellular localization of YAP.^[19]^

## 4. Hippo pathway in orthodontics

Orthodontic tooth movement involves bone resorption on the pressure side and regeneration on the tension side caused by mechanical force. The mechanical loading applied on tooth crown is transmitted to periodontal ligament, which causes changes in extra cellular matrix stiffness, cell shape, and cell stretching.^[20]^

Accumulating evidence suggest that YAP/TAZ play a crucial role in the transduction of mechanical cues into gene expression and cellular response. In a rat tooth movement model, YAP and TAZ expression gradually increased from the second day after force application, peaked on the seventh day, and started to decline from the 14th day. Immunohistochemical staining showed that YAP and TAZ were mainly expressed in osteoblasts, bone matrix, and periodontal ligament cells (PDLCs).^[21]^ Double-labeling immunofluorescence indicated that RUNX2 and TAZ were expressed in the same location; however, their expression pattern was different from that of YAP. The authors suggested that while RUNX2 may function as a direct downstream gene of TAZ to regulate bone remodeling, YAP may regulate tooth movement through its effects on cell proliferation and differentiation.

Wang et al found that mechanical cues can induce TAZ translocation and consequent bone remodeling. They conducted an in vitro experiment where they intermittently applied uniaxial cyclic tensile stress to PDLCs. The results showed that the stress induced the translocation of TAZ into the nucleus of the cells, promoting the expression of osteogenesis-related genes, such as collagen type I, osterix, and osteocalcin. In addition, inhibiting Ras homologue-associated coiled-coil protein kinase signaling was found to suppress the nuclear translocation of TAZ, indicating its regulatory role in this signaling axis.^[22]^ Gu et al discovered that overexpression of TAZ can enhance proliferation and promote osteogenic differentiation of human periodontal ligament stem cells (hPDLSCs) by regulating the downstream molecule SMAD3.^[23]^

As mentioned above, YAP regulates tooth movement through its effects on cell proliferation and differentiation. Jia et al found that overexpressing YAP can promote osteogenic differentiation and inhibit adipogenic differentiation in hPDLSCs by facilitating the nuclear translocation and stabilization of β-catenin.^[23]^ Furthermore, Previous reports have found that downregulating YAP inhibits the proliferation and accelerates the senescence of stem cells from the apical papilla or periodontal ligament.^[24,25]^

## 5. Hippo pathway in periodontal diseases

Periodontal diseases are chronic inflammatory conditions of the tissues surrounding the teeth. When the inflammation is confined to the soft tissues, it manifests as gingivitis. When bone tissue is also affected, it progresses to periodontitis. Severe periodontitis ranked as the sixth-most prevalent health condition worldwide, with approximately 10.8% of the global population suffering from this disease.^[3]^ Periodontitis not only leads to tooth mobility and loss but is also linked to various chronic diseases such as diabetes, cardiovascular diseases, Alzheimer disease, and kidney disease due to shared inflammatory regulatory pathways.^[26–29]^

It is well-known that YAP can transmit mechanical signals, and such effects have bidirectional regulatory roles in periodontitis. For example, Pan et al found in a mouse model of periodontitis that excessive loading forces led to increased local expression of YAP, JNK/AP1, tumor necrosis factor α, and interleukin 6, ultimately resulting in increased bone resorption.^[30]^ On the contrary, under intermittent compressive force, the expression of YAP in PDLCs increased, promoting osteogenic differentiation and inhibiting adipogenic differentiation. When the *YAP* gene was knocked down, the effect of intermittent compressive force was eliminated.^[31]^

Due to excellent regenerative potential, periodontal ligament stem cells hold promise for the treatment of periodontitis. Xiang et al found that calcitonin gene-related peptide could increase the expression of YAP in PDLCs, leading to the upregulation of osteogenesis-related gene expression.^[32]^ Other studies have also made similar discoveries, downstream pathways might involve phosphorylated SMAD3 and Wnt/β-catenin.^[23,33,34]^

To further investigate the therapeutic role of YAP in an inflammatory microenvironment, Dong et al used tumor necrosis factor α to induce inflammation and found that the proliferation and osteogenic differentiation of PDLCs were inhibited. Subsequently, with the overexpression of YAP, the situation improved.^[35]^ This suggests the potential for developing therapeutic strategies targeting YAP. NF-κB signaling pathway might be the potential downstream pathway, as YAP was found to promote the regenerative ability of odontoblasts in an inflammatory environment by inhibiting the NF-κB signaling pathway.^[36]^

## 6. Hippo pathway in dental implant

Tooth loss is one of the significant issues affecting public health and quality of life. The American Association of Oral and Maxillofacial Surgeons reports that among people aged 35 to 44, 70% have lost at least 1 permanent tooth.^[37]^ A meta-analysis reported that the success rate of dental implants can reach as high as 96.4% over a 10-year period.^[38]^ However, implants require a prolonged healing time to achieve osseointegration before they can function properly. Brånemark was the first to propose the concept of osseointegration, describing it as a stable direct contact between bone tissue and the implant. Osseointegration is clinically manifested by the implant being secure and capable of withstanding normal loading. Typically, after osteotomy, the implant is placed, followed by a lengthy bone tissue healing process.

It is well-documented that bone tissue healing involves the coupling of osteogenesis and angiogenesis. Wang et al utilized in vitro experiments to discover that neuropeptides could promote the migration and osteogenic differentiation abilities of mesenchymal stem cells, as well as the angiogenic ability of vascular endothelial cells, by activating YAP. They further verified through animal experiments that neuropeptides could enhance angiogenesis and osteogenesis around implants by upregulating the expression of YAP, thereby promoting rapid healing of the implant.^[39]^ In addition to the local signaling molecules, the surface topography of the implant may influence bone formation. Li et al found that a titanium metal surface with a specific nanotopography could reduce the cytoplasmic YAP through the autophagy-lysosome pathway, thereby promoting the entry of β-catenin into the nucleus and initiating the transcription of osteogenesis-related genes.^[40]^ Interestingly, this regulatory mechanism depended on cell-cell contact. At low cell density, the mechanical signals from the surface morphology could not regulate YAP.

However, recent research indicates that the osseointegration process is not merely a simple healing of bone tissue, but rather an osteoimmune reaction. The host perceives the implant as a foreign body, modulating the local inflammatory response to shield it from the tissues.^[41,42]^ Therefore, the role of immune cells in implant osseointegration is receiving increasing attention. Yuan et al found in animal experiments that manipulating the polarization of macrophages around the implant towards a pro-inflammatory type (M1) and inhibiting the pro-repair type (M2) polarization could affect implant osseointegration.^[43]^ The authors further explored the regulatory mechanisms involved and discovered that Oncostatin M could regulate the direction of macrophage polarization through YAP, ultimately promoting implant osseointegration.^[44]^

## 7. Hippo pathway in tooth extraction

The healing of a tooth extraction socket differs from that of a conventional bone defect in that stem cells from the periodontal ligament may remain within the socket. In addition to the previously discussed effects of the Hippo pathway on PDLCs, some scholars have specifically studied the regulatory role of YAP in the healing of tooth extraction sockets. Cao et al found in a mouse tooth extraction model that mice overexpressing YAP exhibited higher expression levels of bone morphogenetic protein 2, alkaline phosphatase, RUNX2, Osterix, and osteocalcin during the middle and late stages after tooth extraction, increased cell proliferation, and reduced adipogenic differentiation and apoptosis. Micro-computed-tomography analysis revealed better alveolar bone healing in these mice.^[45]^

## 8. Hippo pathway in oral cancer

Because a considerable number of reviews have focused the regulatory role of the Hippo pathway in the development of malignant diseases, we restrict our discussion to the studies on the role of YAP in oral cancer published during the last 3 years.

The known ability of YAP to transmit extracellular mechanical signals led Hasegawa et al to investigate how these external mechanical signals affect intracellular YAP. The results showed that mechanical signals such as cell density and substrate stiffness are conveyed into the cell through Piezo-type mechanosensitive ion channel component 1, activating YAP, and subsequently promoting cell proliferation and tumor growth.^[46]^

In research on therapeutic strategies, several novel molecules have been proposed as potential treatments. Chu et al found that N- (4- (5- (3- (3- (4-acetamido-3-(trifluoromethyl) phenyl) ureido) phenyl) -1,2,4-oxadiazol-3-yl) -3-chlorophenyl) -nicotinamide could act as a novel YAP inhibitor, subsequently inhibiting tumor growth.^[47]^ Moreover, it is reported that Desmoglein-3 inhibits the collective migration of tumor cells by inducing phosphorylation and inactivation of YAP.^[48]^ Furthermore, ribosome Binding Protein 1 regulates cisplatin resistance in oral squamous cell carcinoma through YAP.^[49]^ Lastly, in terms of tumor prognosis research, a retrospective cohort study that examined the distribution of YAP within tumor cells, suggested that the level of YAP in the cell nucleus can be used as a predictive marker for the prognosis of oral squamous cell carcinoma.^[50]^

## 9. Summary

**Figure 2.**
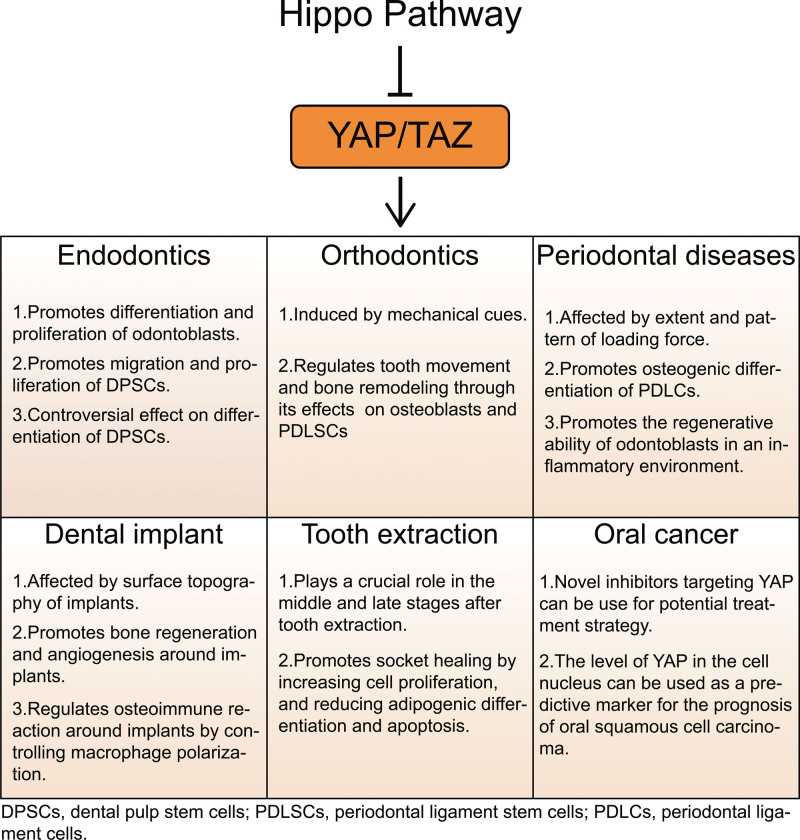
Graphical abstract.

The review discusses the significant role of the Hippo pathway, mainly YAP/TAZ, in various oral health conditions which spans under the scope of various dental specialties such as endodontics, orthodontics, periodontal diseases, dental implantology, tooth extraction, and oral pathology. Figure 2 is the graphical abstract of present review. Endodontics research highlights their role in DPSC differentiation and dentin regeneration. In orthodontics, YAP/TAZ mediate cellular responses to mechanical forces, crucial for tooth movement. In periodontal diseases, YAP/TAZ exhibit bidirectional regulatory roles. In dental implants, they contribute to osseointegration and osteoimmune reactions. The study on tooth extraction sockets focuses on their impact on alveolar bone healing. In oral cancer, recent researches explored YAP’s role in transmitting mechanical signals and developing potential therapeutic strategies. This review underscores the importance of the Hippo pathway in dental medicine, providing insights for future research and potential clinical applications.

## Acknowledgments

We would like to thank Editage (www.editage.com) for English language editing.

## Author contributions

**Conceptualization:** Da Ni.

**Investigation:** Da Ni.

**Methodology:** Da Ni.

**Writing – original draft:** Da Ni.

**Writing – review & editing:** Da Ni.
